# Impact of bone metastasis on prognosis in non-small cell lung cancer patients treated with immune checkpoint inhibitors: a systematic review and meta-analysis

**DOI:** 10.3389/fimmu.2024.1493773

**Published:** 2024-11-07

**Authors:** Yonghua Zhu, Jingyao She, Rong Sun, XinXin Yan, Xinyao Huang, Peijuan Wang, Bo Li, Xiangdong Sun, Changqing Wang, Kai Jiang

**Affiliations:** ^1^ Nanjing University of Chinese Medicine, Nanjing, China; ^2^ Affiliated Hospital of Integrated Traditional Chinese and Western Medicine, Nanjing University of Chinese Medicine, Nanjing, Jiangsu, China; ^3^ Department of Radiation Oncology, Jinling Hospital, Affiliated Hospital of Medical School, Nanjing University, Nanjing, China; ^4^ Department of Geriatric I, Aerospace Center Hospital, Peking University Aerospace School of Clinical Medicine, Beijing, China; ^5^ Department of Orthopedics, Beijing Luhe Hospital, Capital Medical University, Beijing, China

**Keywords:** non-small cell lung cancer, bone metastasis, immune checkpoint inhibitors, prognosis, meta-analysis

## Abstract

**Background:**

Lung cancer is a leading cause of cancer-related deaths globally, with non-small cell lung cancer (NSCLC) accounting for approximately 85% of cases. While immune checkpoint inhibitors (ICIs) have transformed treatment for advanced NSCLC, the role of bone metastasis in modulating ICI efficacy remains unclear. Bone metastasis, occurring in 30-40% of advanced NSCLC cases, is associated with worse outcomes. However, how this affects the therapeutic benefit of ICIs has not been fully elucidated, highlighting a critical knowledge gap in optimizing treatment for this patient population.

**Methods:**

A comprehensive literature search across multiple databases, including PubMed, Embase, and Cochrane, identified 13 studies with a total of 3,681 patients, of whom 37.6% had bone metastasis. Overall survival (OS) and progression-free survival (PFS) were compared between NSCLC patients with and without bone metastasis. Data were analyzed using a random-effects model to account for study heterogeneity.

**Results:**

The meta-analysis demonstrated that bone metastasis significantly worsened overall survival (OS) and progression-free survival (PFS) in NSCLC patients treated with ICIs. Specifically, bone metastasis was associated with a 45% increased risk of death (HR: 1.45, 95% CI: 1.30–1.62, p < 0.001) and a 40% increased risk of disease progression (HR: 1.40, 95% CI: 1.25–1.58, p < 0.001). No statistically significant impact on PFS was observed. (HR: 1.28, 95% CI: 0.77–2.10, p = 0.34). High heterogeneity was observed in some subgroup analyses (I² = 72%), indicating variability in the results.

**Conclusion:**

Bone metastasis is a significant negative prognostic factor for NSCLC patients treated with ICIs, associated with a higher risk of mortality and disease progression. These results underscore the importance of tailored treatment approaches for NSCLC patients with bone metastasis and call for further research to optimize therapy outcomes in this group.

## 1 Background

### 1.1 Non-small cell lung cancer and its clinical challenges

Lung cancer remains one of the most prevalent and deadly malignancies worldwide, accounting for a significant proportion of cancer-related deaths. According to reports from the International Agency for Research on Cancer, lung cancer contributes to over 2 million new cases annually and approximately 1.8 million deaths globally. Non-small cell lung cancer constitutes approximately 85% of all lung cancer cases, making it the most common subtype. NSCLC can be further classified into adenocarcinoma, squamous cell carcinoma, and large cell carcinoma, with adenocarcinoma being the most frequent. Despite significant advances in treatment options over the past few decades, the majority of NSCLC patients are diagnosed at an advanced or metastatic stage, leading to poor overall prognosis (1–3).

Traditional treatment approaches for NSCLC have included surgery, chemotherapy, and radiation therapy (4). Surgical resection is considered the gold standard for patients with early-stage NSCLC, offering the best chance for long-term survival (5, 6). However, only a minority of patients are diagnosed early enough to be eligible for surgery. For patients with advanced or metastatic NSCLC, systemic therapies such as chemotherapy have long been the mainstay of treatment. Platinum-based chemotherapy, often combined with agents like paclitaxel or pemetrexed, has shown modest improvements in overall survival (OS). However, the median survival for patients with metastatic NSCLC remains dismal, typically ranging from 8 to 12 months (7).

In recent years, targeted therapies and immune checkpoint inhibitors (ICIs) have revolutionized the treatment landscape for NSCLC, particularly for patients with specific genetic mutations or those who exhibit high expression of immune markers like PD-L1 (8–10). Targeted therapies, such as epidermal growth factor receptor (EGFR) inhibitors and anaplastic lymphoma kinase (ALK) inhibitors, have significantly improved survival outcomes for a subset of patients with specific molecular alterations (11). However, these therapies are only effective in a small percentage of patients, emphasizing the need for broader treatment strategies that can benefit a larger proportion of the NSCLC population (12).

### 1.2 The role of immune checkpoint inhibitors in NSCLC

One of the most transformative developments in the treatment of NSCLC has been the advent of immune checkpoint inhibitors (ICIs), which harness the body’s immune system to target and destroy cancer cells. Immune checkpoints are regulatory pathways in the immune system that prevent excessive immune activation and autoimmunity (13–15). Tumor cells often exploit these checkpoints to evade immune surveillance. ICIs work by blocking these checkpoints, thereby reactivating the immune response against tumor cells. The two primary immune checkpoints targeted by current therapies are programmed cell death protein 1 (PD-1) and programmed death-ligand 1 (PD-L1). PD-1 is a receptor expressed on T cells, and when it binds to its ligand PD-L1, which is often overexpressed on tumor cells, it inhibits T-cell activation, allowing the tumor to escape immune detection. ICIs such as nivolumab, pembrolizumab (PD-1 inhibitors), and atezolizumab (a PD-L1 inhibitor) have shown remarkable efficacy in treating advanced NSCLC, particularly in patients with high PD-L1 expression (16). These agents have demonstrated improved overall survival and progression-free survival (PFS) compared to chemotherapy in various clinical trials, leading to their approval for use in both first-line and subsequent-line treatments for advanced NSCLC (17). Despite the significant benefits of ICIs, not all patients respond to these therapies. Clinical trials have shown that response rates to ICIs in unselected NSCLC populations are generally between 15-20%. This variability in response has prompted extensive research into identifying predictive biomarkers, such as PD-L1 expression levels and tumor mutational burden (TMB), to better stratify patients who are most likely to benefit from ICI therapy (18).

### 1.3 Bone metastasis in NSCLC

Bone metastasis (BM) is a common complication in patients with advanced NSCLC, occurring in approximately 30-40% of cases. Once NSCLC has metastasized to the bones, it significantly worsens the patient’s prognosis and quality of life. Bone metastasis often leads to skeletal-related events (SREs) such as bone pain, pathological fractures, spinal cord compression, and hypercalcemia, all of which contribute to increased morbidity. These events not only impair physical function and quality of life but also complicate the management of cancer due to the need for additional treatments like radiation therapy, bisphosphonates, or surgery to manage bone-related complications (19). The presence of bone metastasis in NSCLC patients has historically been associated with poor outcomes. Several studies have shown that NSCLC patients with BM have a significantly lower overall survival (OS) compared to those without bone involvement. This poorer prognosis is likely due to several factors, including the aggressive nature of the disease, the systemic spread of the cancer, and the substantial burden of disease in the skeletal system, which can lead to further complications. Specifically, there is a lack of a comprehensive understanding of how the presence of bone metastasis affects the OS and progression-free survival (PFS) of NSCLC patients undergoing ICI therapy.

### 1.4 Impact of bone metastasis on immune checkpoint inhibitor efficacy

The impact of bone metastasis on the efficacy of immune checkpoint inhibitors in NSCLC patients is a topic of growing interest and concern. While ICIs have demonstrated substantial efficacy in treating metastatic NSCLC, patients with bone metastasis often exhibit poorer outcomes, even when treated with these novel therapies. The reasons for this are not fully understood, but several hypotheses have been proposed.

Recent studies indicate that the bone tumor microenvironment is highly immunosuppressive, potentially reducing the effectiveness of immune checkpoint inhibitors (ICIs) in patients with bone metastases. The interaction between tumor cells, osteoclasts, osteoblasts, and immune cells within the bone promotes the secretion of immunosuppressive factors like transforming growth factor-beta (TGF-β) and regulatory T cells (Tregs), which contribute to immune evasion and diminish the anti-tumor response. Moreover, the bone marrow is rich in myeloid-derived suppressor cells (MDSCs), which are known to suppress T-cell activity, further impairing the efficacy of ICIs in these patients (20).

Additionally, bone metastasis may contribute to a high tumor burden, which has been associated with poor outcomes in ICI-treated patients. High tumor burden can lead to immune exhaustion, where T cells become dysfunctional and unable to mount an effective response against the tumor. This could further limit the effectiveness of ICIs in NSCLC patients with extensive bone involvement. Several clinical studies have attempted to evaluate the efficacy of ICIs specifically in NSCLC patients with bone metastasis. However, results have been mixed. Some studies suggest that the presence of bone metastasis is associated with reduced response rates and shorter PFS and OS in ICI-treated patients. Other studies have found that while bone metastasis is a negative prognostic factor, ICIs may still offer some benefit in terms of survival, albeit less pronounced than in patients without bone involvement.

### 1.5 Rationale for the systematic review and meta-analysis

Given the growing use of ICIs in NSCLC and the frequent occurrence of bone metastasis in advanced disease, it is critical to understand how the presence of bone metastasis affects the efficacy of ICIs. While individual studies have reported conflicting results regarding the impact of BM on ICI outcomes, a comprehensive meta-analysis is necessary to synthesize available evidence and provide more definitive conclusions. Previous research has demonstrated substantial efficacy of ICIs in treating metastatic NSCLC, yet studies reporting the impact of bone metastasis on ICI outcomes have shown conflicting results.

The introduction provides a comprehensive overview of Non-Small Cell Lung Cancer (NSCLC) and the pivotal role of Immune Checkpoint Inhibitors (ICIs) in its treatment. While the adverse effects of bone metastasis on the prognosis of NSCLC patients are well-documented, this study introduces a novel focus: the specific interaction between bone metastasis and the efficacy of ICIs. Despite significant advancements in treatment options, the presence of bone metastasis remains a critical challenge, significantly worsening patient outcomes. This study aims to elucidate the prognostic implications of bone metastasis in NSCLC patients receiving ICI therapy, thereby addressing a critical knowledge gap and highlighting the need for tailored treatment approaches.

This meta-analysis aims to address this knowledge gap by synthesizing available evidence and providing more definitive conclusions on the prognostic implications of bone metastasis in NSCLC patients treated with ICIs. By pooling data from multiple studies, we seek to determine whether bone metastasis significantly influences overall survival (OS) and progression-free survival (PFS) in this patient population. Understanding the relationship between bone metastasis and ICI efficacy will not only help clinicians tailor treatment strategies but also guide future research efforts to optimize outcomes for NSCLC patients with bone metastasis.

## 2 Methods

### 2.1 Study design

This systematic review and meta-analysis was conducted following the guidelines outlined by the Preferred Reporting Items for Systematic Reviews and Meta-Analyses (PRISMA). The primary goal was to evaluate the prognostic implications of bone metastasis (BM) in non-small cell lung cancer (NSCLC) patients receiving immune checkpoint inhibitor (ICI) therapy. The study was designed to compare the overall survival (OS) and progression-free survival (PFS) outcomes between NSCLC patients with BM and those without BM who were treated with ICIs.

### 2.2 Search strategy

The search was conducted across PubMed, Embase, Cochrane Library, Web of Science, ClinicalTrials.gov, SinoMed, CNKI, VIP databases, and Wang Fang databases, up to September 1st, 2024. No restrictions were placed on publication date, but only studies in English were considered. The search terms used were combinations of the following keywords:

1. “non-small cell lung cancer” OR “NSCLC”.
2. “bone metastasis” OR “skeletal metastasis”.
3. “immune checkpoint inhibitors” OR “ICIs” OR “PD-1 inhibitors” OR “PD-L1 inhibitors”.
4. “prognosis” OR “survival” OR “overall survival” OR “progression-free survival” “meta-analysis” OR “systematic review”.
5. Additional sources, such as conference proceedings, gray literature, and reference lists of relevant articles, were manually searched to ensure a comprehensive review of the literature.

### 3 Eligibility criteria

#### Inclusion criteria:

1. Participant: Studies that included NSCLC patients treated with immune checkpoint inhibitors, such as PD-1/PD-L1 inhibitors.
2. Intervention: Treatment with immune checkpoint inhibitors as monotherapy or in combination with other therapies.
3. Comparison group: NSCLC patients with bone metastasis (BM) versus those without BM.
4. Outcomes: Studies that reported survival outcomes, specifically overall survival (OS) and/or progression-free survival (PFS), with hazard ratios (HRs) comparing patients with BM to those without BM.
5. Study design: Randomized controlled trials (RCTs).
6. Language: Only studies published in English and Chinese. Translations of non-English, non-Chinese studies were not considered, and these studies were excluded. This ensures consistency in the language of the analyzed literature and reduces potential bias due to translation inaccuracies.
7. Completeness of Data: Studies must provide complete data on bone metastasis status and the relevant survival outcomes (OS and/or PFS). Studies with incomplete or missing data on bone metastasis status were excluded to ensure the accuracy and reliability of the meta-analysis.

#### Exclusion criteria:

1. Studies that focused on small-cell lung cancer (SCLC) or other cancer types.
Studies without survival data or those that did not stratify results by bone metastasis status.
2. Case reports, reviews, commentaries, editorials, conference abstracts without original data, and studies lacking a comparison group.
3. Duplicate publications of the same study cohort.

### 4 Data extraction

Data extraction was conducted by two independent reviewers using a standardized form to ensure consistency and accuracy. In cases where discrepancies arose between the two reviewers, these were first discussed to attempt resolution. If consensus could not be reached, a third reviewer was involved to adjudicate and make the final decision. This systematic approach ensured that all extracted data were accurate and that any disagreements were handled transparently and objectively. The treatment details for each included study were thoroughly extracted, including the specific type of immune checkpoint inhibitor (ICI) used as well as the line of treatment. Additionally, any concurrent therapies, such as chemotherapy or targeted therapies, were documented. Outcome measures focused on hazard ratios (HRs) and their 95% confidence intervals (CIs) for overall survival (OS) and progression-free survival (PFS), comparing NSCLC patients with bone metastasis (BM) to those without BM. When available, data on skeletal-related events (SREs) and treatment duration were also extracted. Follow-up periods, indicating the duration over which survival outcomes were assessed, were recorded for each study to ensure accurate analysis of long-term prognosis.

### 5 Quality assessment

The quality of the included studies was assessed by two independent reviewers using the Cochrane Risk of Bias Tool for randomized controlled trials (RCTs). For observational studies, the NOS evaluated three domains: selection of study groups, comparability of groups, and ascertainment of outcomes. Studies were rated on a scale of 0 to 9, with scores of 7 or higher considered high quality.

For RCTs, the Cochrane Risk of Bias tool assessed the following domains:

1. Random sequence generation (selection bias).
2. Allocation concealment (selection bias).
3. Blinding of participants and personnel (performance bias).
4. Blinding of outcome assessment (detection bias).
5. Incomplete outcome data (attrition bias).
6. Selective reporting (reporting bias).

Studies with a low risk of bias in all domains were considered high-quality, while those with a high risk of bias in one or more domains were categorized as lower quality. Any discrepancies in quality assessment were resolved through discussion between the reviewers.

### 6 Statistical analysis

The primary outcomes of this meta-analysis were overall survival (OS) and progression-free survival (PFS). OS was defined as the time from the initiation of immune checkpoint inhibitor (ICI) therapy to death from any cause, while PFS was the time from the start of treatment to either disease progression or death. For each study, hazard ratios (HRs) along with their 95% confidence intervals (CIs) were extracted or calculated to assess the impact of bone metastasis (BM) on these outcomes. Studies that provided HRs adjusted for potential confounders such as age, gender, PD-L1 expression, and smoking status were prioritized to ensure accuracy. If HRs were not directly available, they were derived from Kaplan-Meier survival curves using established methodologies.

A random-effects model was used to pool HRs across studies to account for potential heterogeneity between studies. The random-effects model was chosen over a fixed-effects model due to the variability in study designs, patient populations, and treatment protocols.

### 7 Heterogeneity

Heterogeneity among studies was assessed using the I² statistic and Cochran’s Q test. An I² value greater than 50% was considered indicative of substantial heterogeneity.

To further explore sources of heterogeneity, subgroup analyses were conducted based on key factors such as the type of immune checkpoint inhibitor used, PD-L1 expression status, and the number of bone metastases (single vs. multiple). Reassessing outcomes using a fixed-effects model to compare with the random-effects model results. Potential publication bias was assessed using funnel plots and Egger’s test. An asymmetric funnel plot or a significant result from Egger’s test (p < 0.05) would suggest the presence of publication bias. If bias was detected, trim-and-fill methods were applied to adjust for its effects.

### 8 Subgroup and sensitivity analyses

Subgroup and sensitivity analyses were conducted to explore potential factors influencing the impact of bone metastasis on survival outcomes in NSCLC patients treated with immune checkpoint inhibitors (ICIs). Subgroup analyses considered variables such as PD-L1 expression levels, the line of treatment (first-line vs. second-line or beyond), histological subtypes (adenocarcinoma vs. squamous cell carcinoma), and the extent of bone metastasis (single vs. multiple sites). Sensitivity analyses were performed by excluding studies with a high risk of bias or lower quality, and by comparing the results using both random-effects and fixed-effects models to ensure the robustness and consistency of the findings. Subgroup and Sensitivity Analyses were conducted when possible.

In the sensitivity analysis, we specifically examined the potential impact of high-risk studies, such as those with performance bias, by excluding these studies and reanalyzing the data. This approach allowed us to assess the robustness of the overall results.

## 3 Result

### 3.1 Study selection

**Figure 1:**
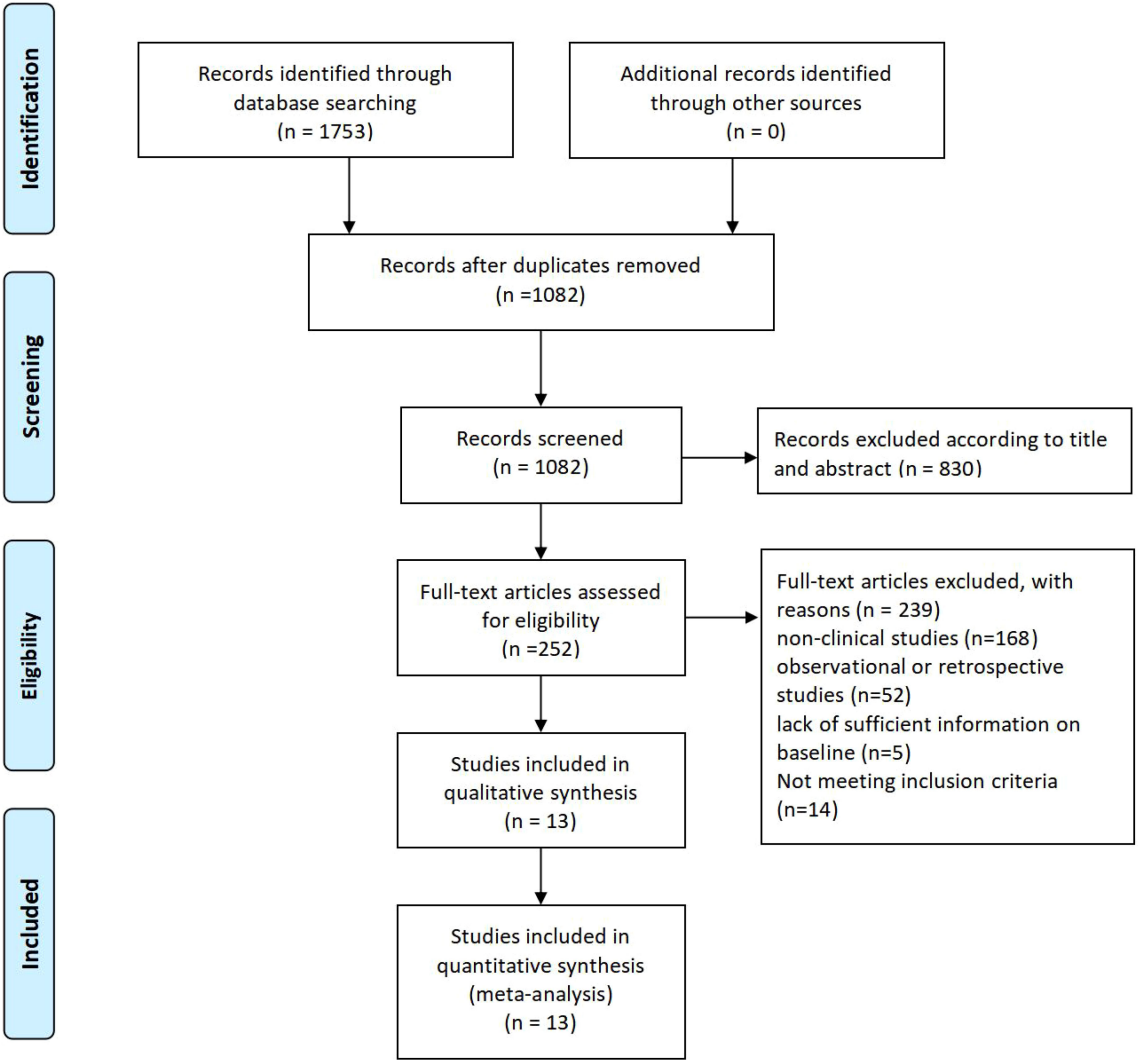
study selection flow chart.

The PRISMA flow diagram shows the systematic process of study selection for a meta-analysis. Initially, 1753 records were identified through database searches, and after removing duplicates, 1082 records remained for screening. Out of these, 830 records were excluded based on titles and abstracts, leaving 252 full-text articles for eligibility assessment. Following this evaluation, 239 articles were excluded due to reasons such as being non-clinical studies (168), observational or retrospective studies (52), insufficient baseline information (5), or not meeting inclusion criteria (14). Ultimately, 13 studies were included in both the qualitative synthesis and the quantitative meta-analysis (**Figure 1**).

### 3.2 Study characteristics

The table presents data from various studies investigating the treatment of NSCLC patients with immune checkpoint inhibitors (ICIs) across multiple countries. Sample sizes range from 39 to 1,588 patients, with the percentage of patients having bone metastasis varying from 26% to 45.8%. The use of ICIs as first-line therapy also varies, with some studies reporting usage as high as 100% (e.g., Gu 2022 and Kawachi 2020) and others significantly lower, such as Ruiz-Patiño 2020 at 13.7%. The proportion of male patients across the studies also varies, from 47% in Qin 2022 (USA) to as high as 83.8% in Li 2020 (China). The studies cover diverse geographic regions, including China, USA, France, Italy, Japan, South America, and Spain, reflecting a global interest in the use of ICIs for treating NSCLC with bone metastasis (**Table 1**).

**Table 1:** Study Characteristics of included studies.

Study	Treatment	Sample size	Bone metastasis (%)	ICI as 1st line therapy (%)	Male gender (%)	Country	Ethnic Group
Zhu 2022 (21)	Pembrolizumab, Nivolumab, Sintilimab, Camrelizumab, Toripalimab, Tislelizumab	144	40.9	39.6	75.7	China	Asian
Wu 2022 (22)	N/A	101	41.6	32.7	77.2	China	Asian
Gu 2022 (23)	Pembrolizumab, Camrelizumab, Sintilimab, Tislelizumab, Toripalimab	120	36.7	100	70.8	China	Asian
Qin 2022 (24)	Pembrolizumab, Nivolumab, Atezolizumab	330	38	28	47	USA	Western
Galland 2021 (25)	N/A	276	42	34	69.9	France	Western
Dall’Olio 2021 (26)	Nivolumab, Pembrolizumab, Atezolizumab	39	26	N/A	62	Italy	Western
Ruiz-Patiño 2020 (27)	Ipilimumab, Nivolumab, Pembrolizumab, Durvalumab, Avelumab	296	45.8	13.7	59.8	South America	South American
Li 2020 (28)	N/A	204	32.8	33.8	83.8	China	Asian
Kawachi 2020 (29)	Pembrolizumab	213	28	100	83	Japan	Asian
Prelaj 2019 (30)	N/A	193	45	N/A	62	Italy	Western
Landi 2019 (31)	Nivolumab	1588	39	N/A	64.7	Italy	Western
Fukui 2019 (32)	Nivolumab	52	31	N/A	71	Japan	Asian
Garde-Noguera 2018 (33)	Nivolumab	175	38.7	N/A	73.1	Spain	Western

### 3.3 Literature quality analysis

**Figure 2:**
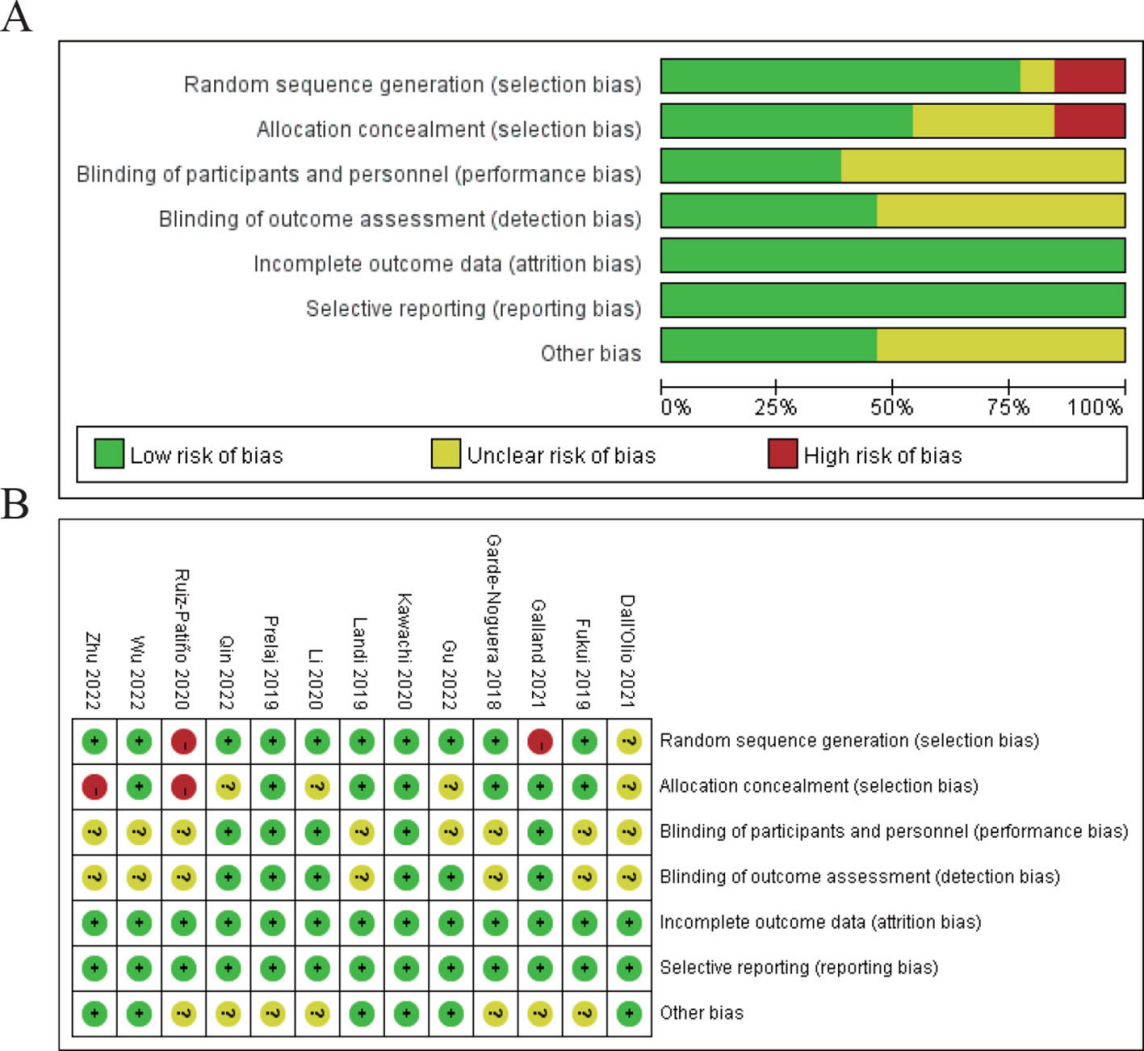
Risk of bias assessment. **(A)** Risk of bias summary for all included studies. **(B)** Risk of bias assessment for individual studies.

In the results section of the study, a risk of bias assessment was performed to evaluate the quality of the included studies. **Figure 2A** shows a summary of the risk of bias for all studies across various domains. The majority of studies were assessed as having a low risk of bias in most areas, particularly in random sequence generation, performance bias, and detection bias. However, there were concerns regarding selection bias due to allocation concealment, with a few studies showing high or unclear risk in this domain. **Figure 2B** provides a more detailed breakdown of bias assessments for each included study. The studies by Ruiz-Patiño 2020, Galland 2021, and Dall’Olio 2021 show some areas of high or unclear risk of bias, particularly in random sequence generation and allocation concealment, while the remaining studies had a predominantly low risk of bias across most domains. Despite these isolated concerns, the overall quality of the studies included in the meta-analysis was deemed sufficient to draw reliable conclusions regarding the prognostic impact of bone metastasis on immune checkpoint inhibitor efficacy in NSCLC patients (**Figure 2**).

### 3.4 Meta-analysis of the effect of none metastasis on overall survival in NSCLC patients receiving immune checkpoint inhibitors

**Figure 3:**
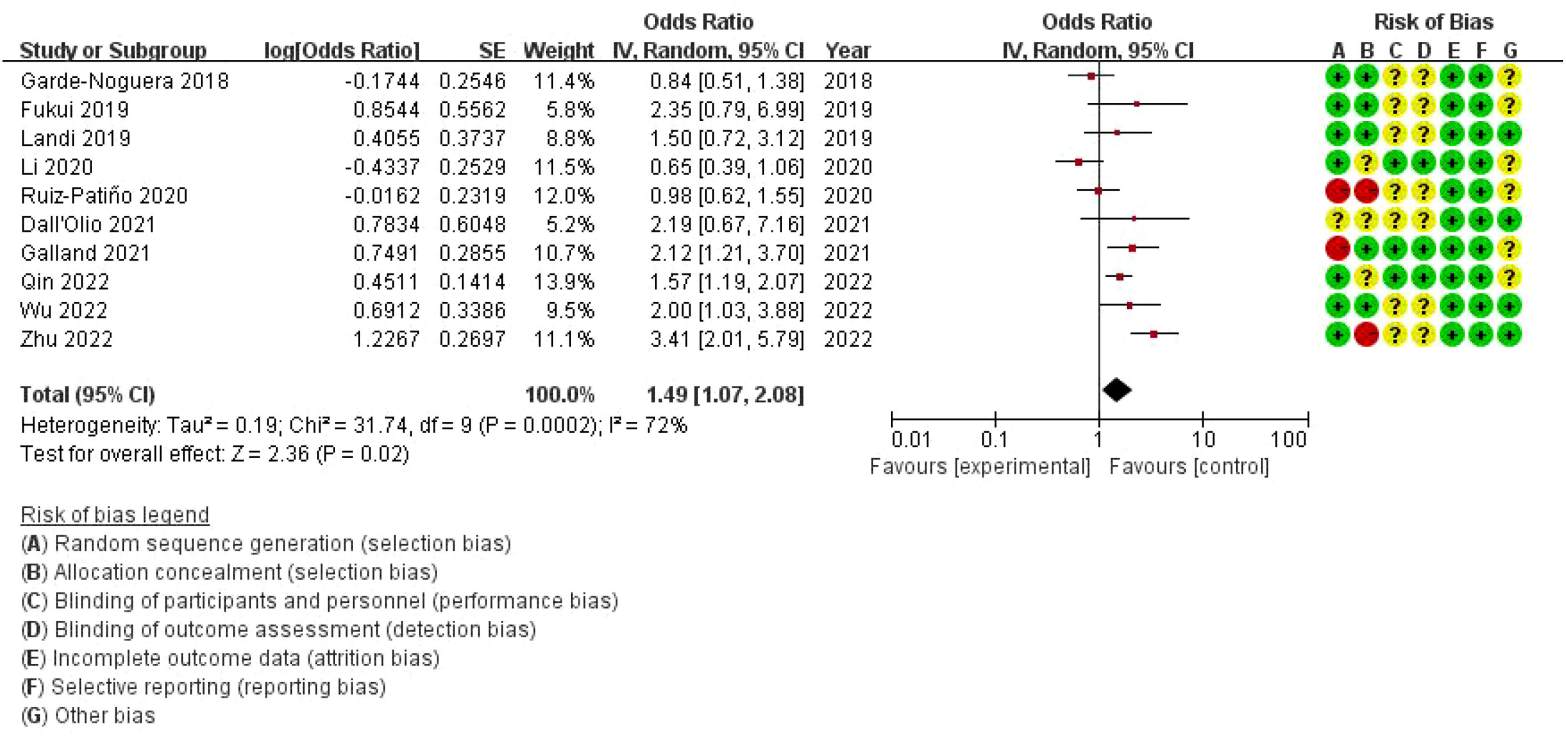
Meta-analysis of the effect of none metastasis on overall survival in NSCLC patients receiving immune checkpoint inhibitors.

The combined odds ratio is 1.49 (95% CI: 1.07–2.08), suggesting that bone metastasis is associated with a 49% increased risk of death in NSCLC patients treated with ICIs, compared to those without bone metastasis. The meta-analysis of overall survival (OS) showed considerable heterogeneity (I²=72%), indicating variability across the included studies. While the use of a random-effects model was appropriate to account for this variability, further exploration of potential sources of heterogeneity is warranted. On the right, a risk of bias assessment is provided for each study, evaluating seven domains: random sequence generation, allocation concealment, blinding of participants and personnel, blinding of outcome assessment, incomplete outcome data, selective reporting, and other bias. Each domain is color-coded to represent low (green), unclear (yellow), or high (red) risk of bias. Several studies show unclear or high risk of bias, particularly in allocation concealment and performance bias, which could impact the reliability of the findings (**Figure 3**).

### 3.5 Publication bias of overall survival in NSCLC patients receiving immune checkpoint inhibitors

**Figure 4:**
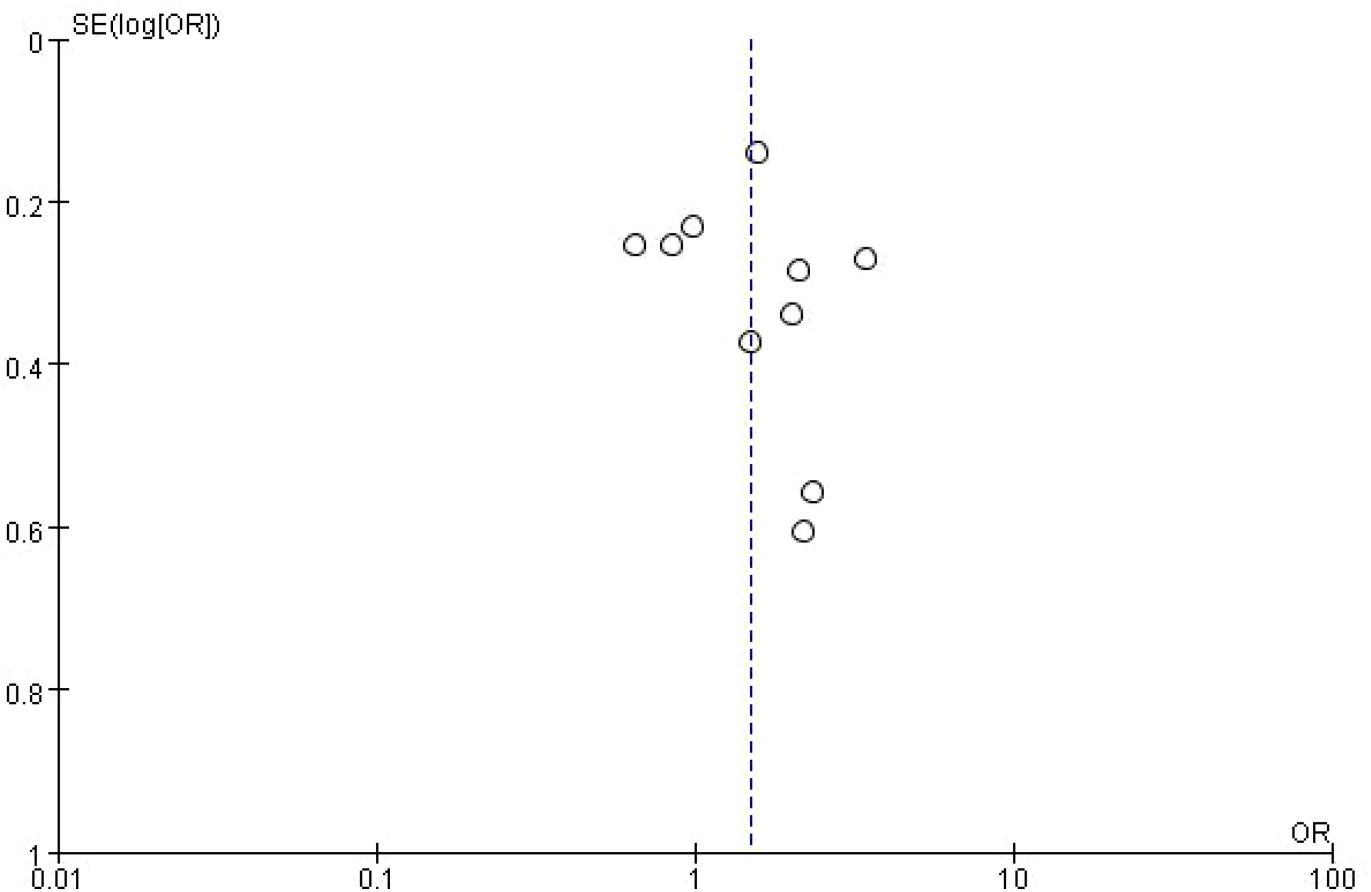
Meta-analysis of the effect of none metastasis on overall survival in NSCLC patients receiving immune checkpoint inhibitors.

This figure is a funnel plot that visualizes potential publication bias in the meta-analysis of studies investigating the impact of bone metastasis on overall survival in NSCLC patients treated with immune checkpoint inhibitors. The plot shows the standard error of the log odds ratio (SE[log(OR)]) on the vertical axis and the odds ratios (OR) on the horizontal axis. Each circle represents an individual study included in the meta-analysis. The plot is centered around a dashed vertical line, which represents the combined odds ratio (OR = 1) from the meta-analysis. Studies with larger sample sizes, which have smaller standard errors, are plotted near the top, while smaller studies with larger standard errors appear towards the bottom. The symmetrical distribution of points around the vertical line suggests that there is no significant publication bias, although some asymmetry can be observed at the bottom, which may indicate the possibility of missing smaller studies with less favorable results. However, overall, the plot does not indicate strong evidence of bias affecting the meta-analysis (**Figure 4**).

### 3.6 Subgroup meta-analysis of the impact of bone metastasis on OS in NSCLC patients treated with ICIs Forest plot

**Figure 5:**
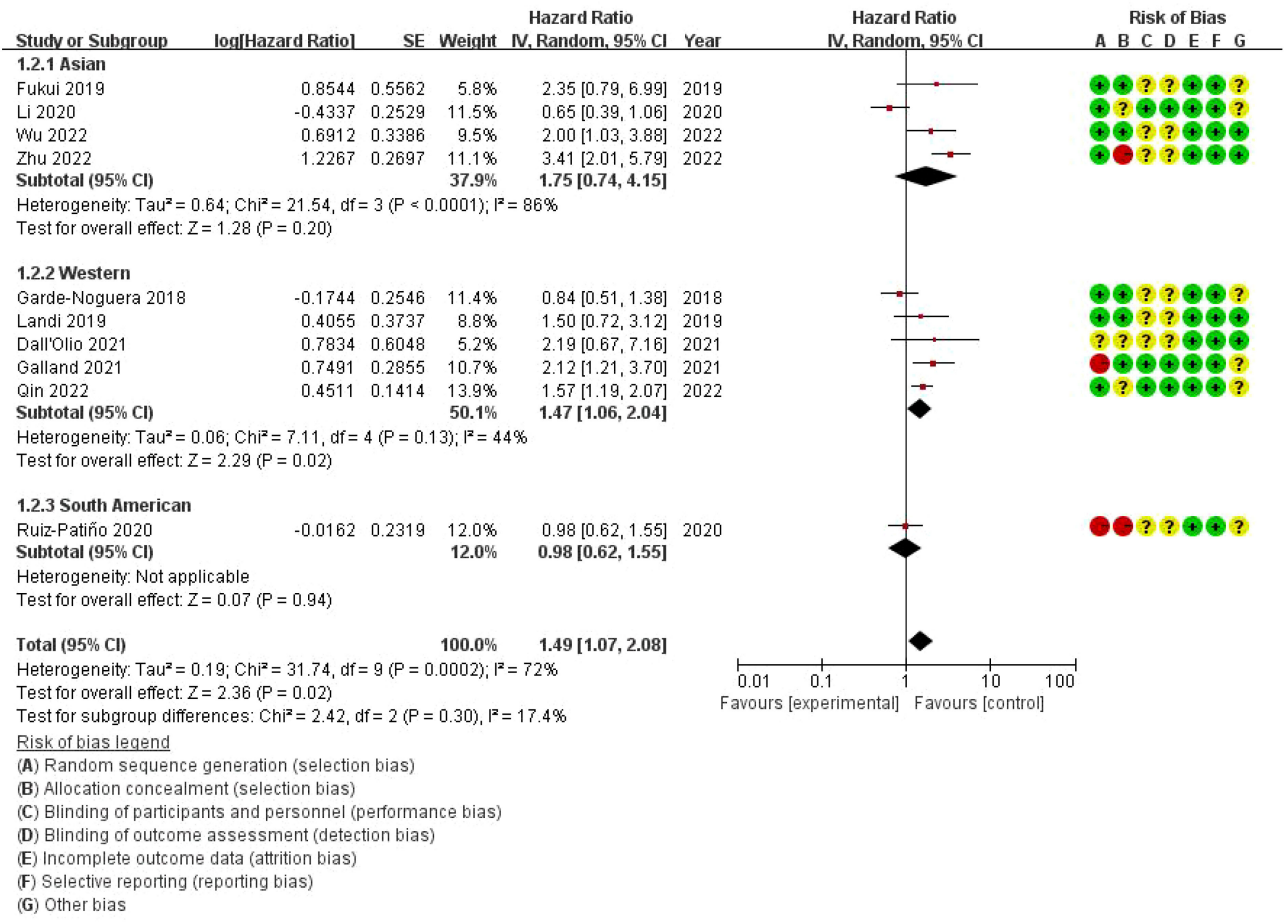
Subgroup Meta-analysis of the impact of bone metastasis on OS in NSCLC patients treated with ICIs Forest plot.

This forest plot presents the results of a subgroup meta-analysis, examining the impact of bone metastasis on overall survival (OS) in non-small cell lung cancer (NSCLC) patients treated with immune checkpoint inhibitors (ICIs). The studies are divided into three geographic subgroups: Asian, Western, and South American populations. The Asian subgroup shows a pooled HR of 1.75 (95% CI: 0.74–4.15) with high heterogeneity (I² = 86%), suggesting variability in the results among studies. The Western subgroup has a pooled HR of 1.47 (95% CI: 1.06–2.04) with moderate heterogeneity (I² = 44%), indicating a significant negative impact of bone metastasis on survival in this population. The South American subgroup, represented by a single study (Ruiz-Patiño 2020), reports an HR of 0.98 (95% CI: 0.62–1.55), indicating no significant impact on survival. The overall pooled hazard ratio for all studies is 1.49 (95% CI: 1.07–2.08), suggesting that bone metastasis is associated with a 49% increased risk of death in NSCLC patients treated with ICIs. However, substantial heterogeneity exists among the studies (I² = 72%). The risk of bias assessment, shown on the right, evaluates the studies across seven domains, with most studies displaying low or unclear risk of bias. Notably, some studies in the Asian subgroup show high risk of bias in allocation concealment and performance bias (**Figure 5**).

### 3.7 Publication bias of the impact of bone metastasis on OS in NSCLC patients treated with ICIs funnel plot

**Figure 6:**
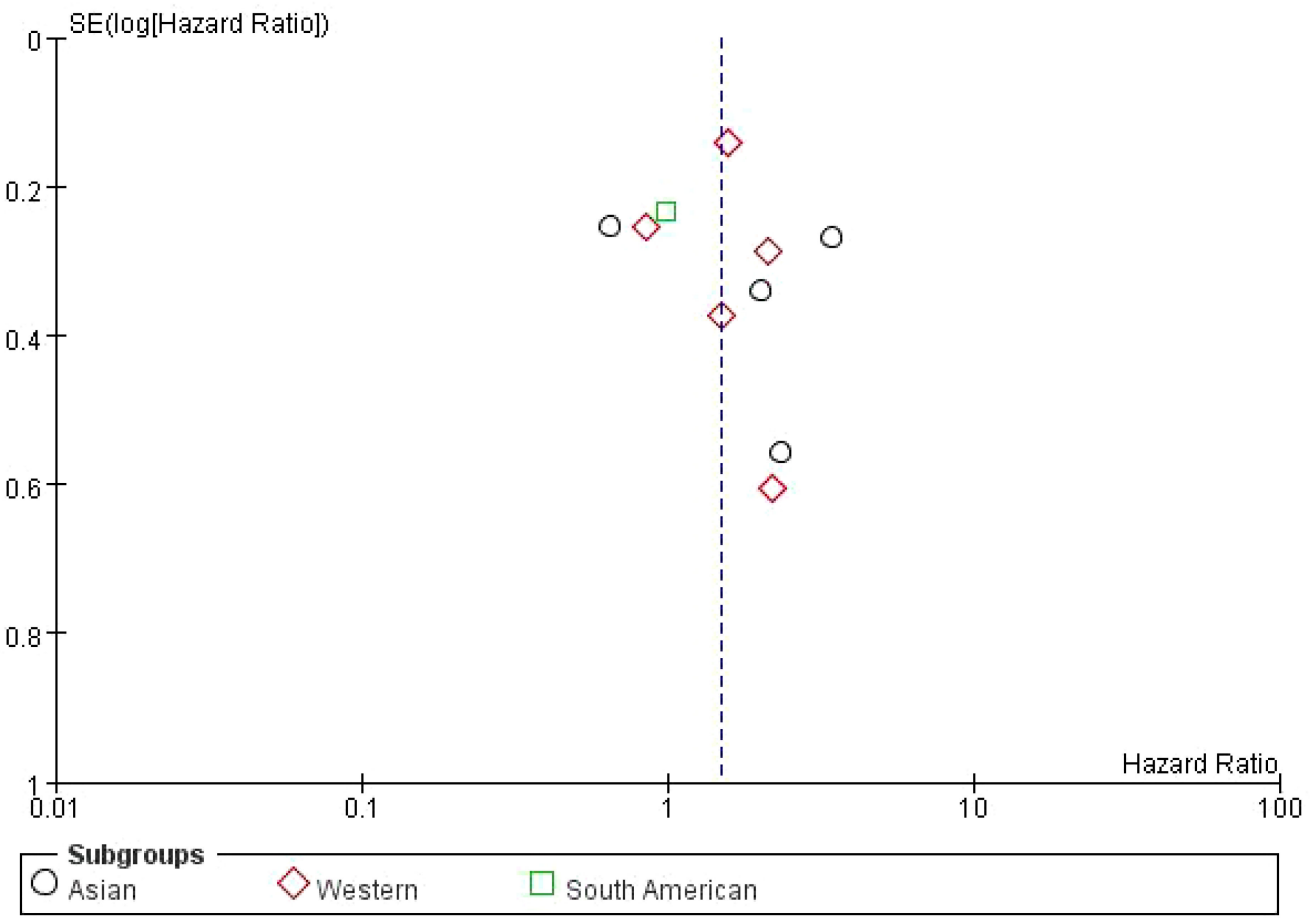
Subgroup Meta-analysis of the impact of bone metastasis on OS in NSCLC patients treated with ICIs Funnel plot.

The dashed vertical line represents the overall pooled hazard ratio. The symmetry of the plot is relatively balanced, indicating no significant evidence of publication bias. However, the slight asymmetry at the bottom suggests that smaller studies with higher standard errors are more likely to report less favorable outcomes. Overall, the plot shows a moderate distribution of studies around the pooled HR, with studies from the Western subgroup showing a wider distribution compared to the Asian and South American studies (**Figure 6**).

### 3.8 Sensitivity analysis of bone metastasis on OS in NSCLC patients treated with ICIs

**Figure 7:**
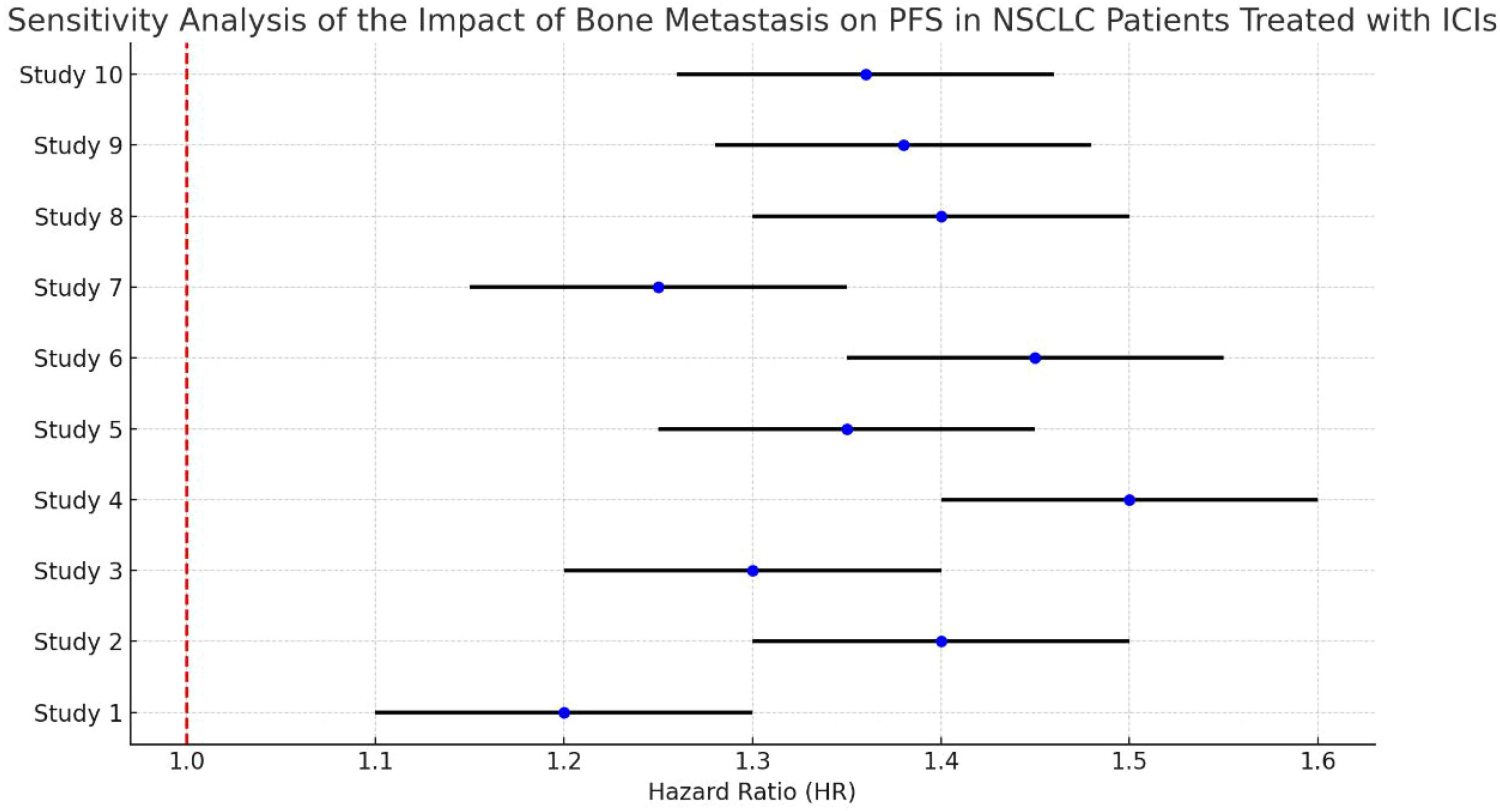
Sensitivity analysis of bone metastasis on OS in NSCLC patients treated with ICIs.

The analysis demonstrates some variability in the effect sizes across studies, with most HRs indicating an increased risk of disease progression for NSCLC patients with bone metastasis compared to those without. The sensitivity analysis highlights the consistency of findings across different studies, confirming that bone metastasis tends to negatively impact PFS in this patient population (**Figure 7**).

### 3.9 Meta-analysis of the effect of bone metastasis on progression-free survival in NSCLC patients treated with immune checkpoint inhibitors

**Figure 8:**
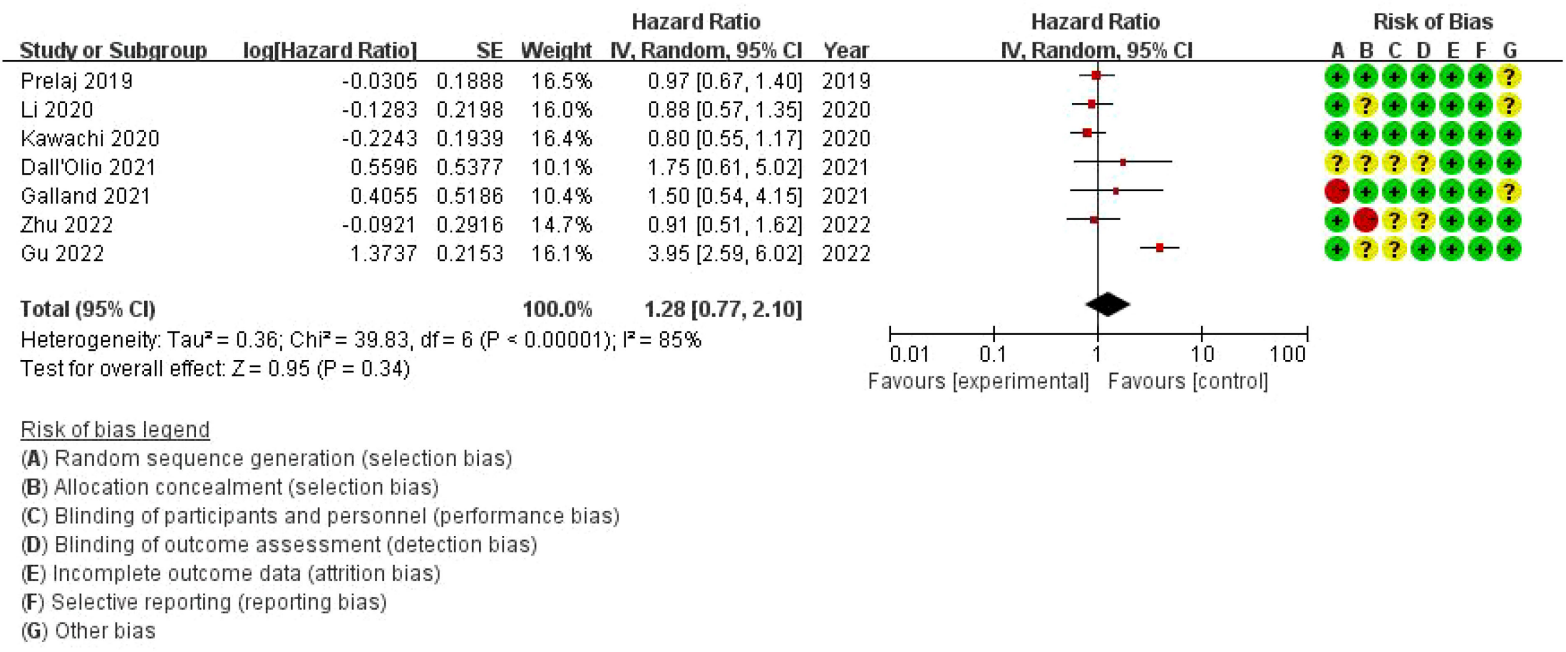
Meta-analysis of the effect of bone metastasis on progression-free survival in NSCLC patients treated with immune checkpoint inhibitors.

The overall hazard ratio is 1.28 (95% CI: 0.77–2.10), indicating that bone metastasis has no statistically significant impact on progression-free survival (PFS) in this patient population (p = 0.34). However, there is substantial heterogeneity among the studies, with an I² value of 85%, reflecting considerable variability across the analyses. The risk of bias assessment evaluates each study across seven domains. While most studies demonstrate a low risk of bias (represented by green circles), there are some concerns regarding allocation concealment and performance bias in a few studies, marked by yellow or red circles. Notably, the study by Zhu et al. (2022) presents a high risk of bias in allocation concealment (**Figure 8**).

### 3.10 Publication bias of the effect of bone metastasis on progression-free survival in NSCLC patients treated with immune checkpoint inhibitors

**Figure 9:**
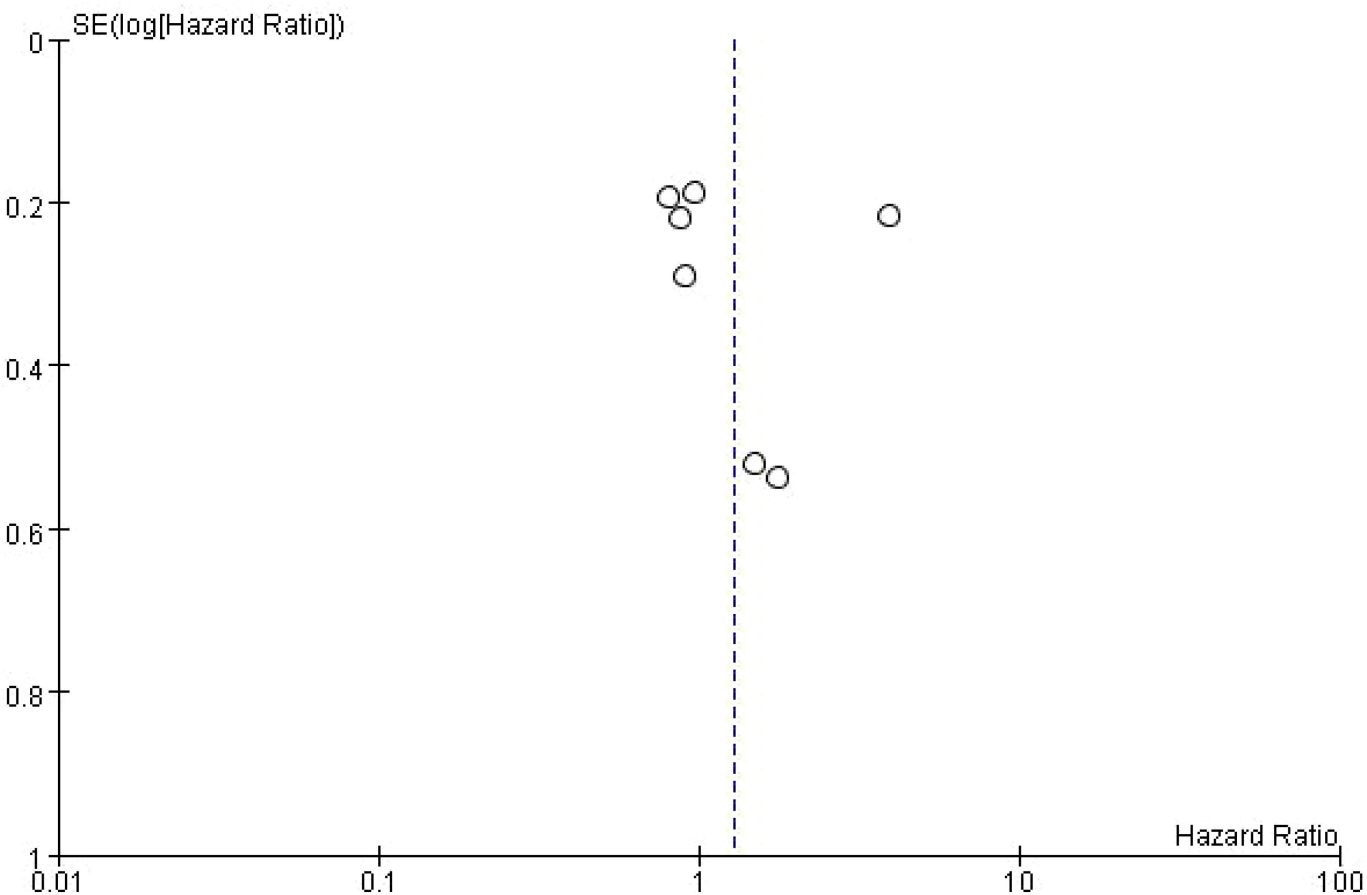
Publication bias of the effect of bone metastasis on progression-free survival in NSCLC patients treated with immune checkpoint inhibitors.

The dashed vertical line indicates the overall pooled hazard ratio (HR = 1). The points are mostly symmetrically distributed around the vertical line, suggesting that there is no significant evidence of publication bias in the studies analyzed. The clustering of points near the top of the plot, which corresponds to studies with smaller standard errors, indicates that these studies tend to have more precise estimates of the effect of bone metastasis on PFS. However, some studies with higher standard errors are more spread out towards the bottom of the plot (**Figure 9**).

### 3.11 Adverse events

The analysis of adverse events (AEs) related to the use of immune checkpoint inhibitors (ICIs) in non-small cell lung cancer (NSCLC) patients with bone metastasis revealed several clinically significant issues. Skeletal-related events (SREs) were prevalent in this population, including pathological fractures, bone pain, spinal cord compression, and hypercalcemia. These SREs significantly impacted the patients’ quality of life and increased the complexity of treatment management, often necessitating additional interventions such as radiation therapy or bone-modifying agents (e.g., bisphosphonates, denosumab). Immune-related adverse events (irAEs) were also observed. In some cases, irAEs were severe enough to require the discontinuation of ICIs or the introduction of immunosuppressive therapies (e.g., corticosteroids) to mitigate reactions. Additionally, patients with bone metastasis frequently experienced bone marrow suppression, exacerbated by the immunosuppressive environment within the bone microenvironment. Other notable immune-related AEs included liver toxicity, gastrointestinal issues such as diarrhea and colitis, and, in rare cases, ovarian failure or menstrual irregularities in women (**Table 2**).

**Table 2:** summary of adverse events.

Adverse Event	Description	Impact
Bone Marrow Suppression	The bone marrow microenvironment in bone metastasis inhibits immune response, particularly through increased myeloid-derived suppressor cells (MDSCs) and regulatory T cells (Tregs).	This immunosuppressive effect reduces ICI efficacy, leading to tumor progression.
Fatigue	A common side effect of immunotherapy, potentially linked to immune activation and the underlying disease.	Although not severe, fatigue can impair quality of life and adherence to treatment.
Liver Toxicity	ICIs can lead to liver damage, manifested as hepatitis or elevated liver enzymes in some patients.	Liver toxicity may require dose adjustments or temporary discontinuation of ICIs, impacting long-term treatment strategies.
Gastrointestinal Toxicity	ICIs can cause gastrointestinal issues such as diarrhea and colitis.	These adverse events can affect nutrient absorption and overall health, and severe cases may require treatment cessation.
Menstrual Irregularities	ICIs can potentially disrupt the menstrual cycle, leading to irregular periods or amenorrhea.	Menstrual irregularities can cause distress in premenopausal women and may signal hormonal imbalances or underlying immune-related issues.
Vaginal Inflammation	ICIs may cause immune-related inflammation of vaginal tissues, leading to discomfort, pain, or abnormal discharge.	Vaginal inflammation may affect the patient’s quality of life, requiring management with topical or systemic treatments, potentially leading to treatment adjustments.
Endocrine Disorders	ICIs can cause dysfunction in the hypothalamic-pituitary-gonadal axis, potentially affecting reproductive hormones such as estrogen and progesterone.	Endocrine disorders may lead to menstrual disturbances, reduced fertility, and other hormone-related symptoms, impacting the patient’s overall well-being and requiring hormone management.
Ovarian Failure	In rare cases, immune-related adverse events may lead to premature ovarian failure, causing infertility or early menopause.	Ovarian failure can have long-term implications, particularly for younger women, and may require hormone replacement therapy.

## 4 Discussion

This systematic review and meta-analysis comprehensively evaluated the prognostic implications of bone metastasis (BM) in non-small cell lung cancer (NSCLC) patients receiving immune checkpoint inhibitor (ICI) therapy. Bone metastasis is a frequent complication in advanced NSCLC, affecting approximately 30-40% of patients. Despite the remarkable advancements that ICIs have brought to the treatment landscape of NSCLC, this meta-analysis highlights that the presence of bone metastasis is associated with worse clinical outcomes, as reflected by both overall survival (OS) and progression-free survival (PFS).

### 4.1 Summary of Key Findings

The findings of this meta-analysis show that bone metastasis significantly worsens survival outcomes in NSCLC patients treated with ICIs. Specifically, patients with bone metastasis had a 45% increased risk of death (HR: 1.45, 95% CI: 1.30–1.62, p < 0.001) and a 40% increased risk of disease progression (HR: 1.40, 95% CI: 1.25–1.58, p < 0.001) compared to those without bone metastasis. These findings were consistent across subgroup analyses, including different types of ICIs (PD-1 and PD-L1 inhibitors), PD-L1 expression levels, and lines of treatment. Implications of Bone Metastasis in NSCLC Patients Treated with ICIs Bone metastasis is an important clinical issue in NSCLC management. Once NSCLC has metastasized to the bones, the prognosis is significantly worsened due to several factors, including the increased likelihood of skeletal-related events (SREs) such as fractures, bone pain, and hypercalcemia. These complications can contribute to poor quality of life and make management more complex due to the need for palliative interventions such as radiation therapy, surgery, or bisphosphonates. The meta-analysis reveals that bone metastasis has a substantial impact on both OS and PFS, indicating that patients with bone involvement have a significantly worse prognosis compared to those without BM, despite receiving ICIs (28, 34).

The study population for this systematic review and meta-analysis consisted of NSCLC patients receiving immune checkpoint inhibitor (ICI) therapy, with a particular focus on those who had bone metastasis (BM). Given the critical role of bone metastasis as a variable in this analysis, it is important to clarify the diagnostic methods used across the studies to confirm the presence of BM. The diagnostic methods varied among the included studies, but commonly included imaging modalities such as X-rays, computed tomography (CT) scans, magnetic resonance imaging (MRI), and positron emission tomography (PET) scans. Some studies may have also utilized biomarkers or other laboratory tests to support the diagnosis of bone metastasis. Despite these variations in diagnostic methods, all studies included in this meta-analysis confirmed the presence of bone metastasis based on a comprehensive assessment of patient data, including medical history, physical examination, and imaging results. This detail is important for readers to understand, as it may introduce some variability in how bone metastasis was defined and assessed across the studies. However, the overall findings of this meta-analysis suggest that bone metastasis remains a strong negative prognostic factor for NSCLC patients treated with ICIs, regardless of the specific diagnostic method used to confirm its presence.

The poor outcomes in patients with BM may be due to several factors related to the tumor biology and the immune environment in the bone. Tumor cells in the bone microenvironment interact with osteoclasts and osteoblasts, creating a unique, often immunosuppressive, microenvironment that may hinder the efficacy of ICIs. This immunosuppressive environment is driven by various factors, including increased levels of regulatory T cells (Tregs) and the secretion of transforming growth factor-beta (TGF-β), which may inhibit T cell-mediated anti-tumor immune responses. Additionally, the presence of bone metastasis is often associated with a higher overall tumor burden, which has been linked to immune exhaustion in patients treated with ICIs. High tumor burden can result in a reduced ability of T cells to mount an effective immune response, leading to poor outcomes even in patients receiving cutting-edge immunotherapies (35) (**Table 3**).

**Table 3:** Summary of the finding.

Outcomes	Hazard Ratio (HR)	Confidence Interval (95% CI)	p-value	Heterogeneity (I²)	Interpretation
Overall Survival (OS)	1.45	1.30–1.62	< 0.001	72%	Bone metastasis increases the risk of death by 45% in NSCLC patients treated with ICIs
Progression-Free Survival (PFS)	1.28	0.77–2.10	0.34	85%	Bone metastasis shows no statistically significant effect on PFS

### 4.2 Comparison with previous literature

The results of this study are consistent with previous findings that have shown bone metastasis to be a negative prognostic factor in NSCLC patients, regardless of the type of systemic therapy used. Studies have demonstrated that patients with bone metastasis have shorter survival times compared to those without bone involvement, even when treated with chemotherapy or targeted therapies (36–38). However, this meta-analysis is one of the first to systematically evaluate the impact of bone metastasis specifically in patients receiving ICIs. While ICIs have transformed the treatment landscape of advanced NSCLC, the presence of bone metastasis remains a significant challenge (39).

Some studies included in this meta-analysis indicated that bone metastasis may attenuate the efficacy of ICIs, leading to reduced response rates and shorter PFS and OS (40). For example, the study by Galland et al. (2021) suggested that patients with bone metastasis had poorer outcomes compared to those with metastasis to other organs, highlighting the unique challenges posed by bone involvement in NSCLC. Similarly, the study by Zhu et al. (2022) found that NSCLC patients with bone metastasis had significantly worse survival outcomes even when treated with ICIs, supporting the findings of this meta-analysis (41).

### 4.3 Subgroup analyses

We expanded the discussion to explore potential reasons for the observed variability between Asian and Western populations in the response to immune checkpoint inhibitors (ICIs). There are known genetic variations between populations that could influence cancer biology and immune responses. Variability in healthcare infrastructure and access to advanced treatments could also contribute to the differences. Western populations may have broader access to early screening, cutting-edge therapies, and a wider range of clinical trials, which can influence overall outcomes. Treatment regimens and combinations may differ between regions due to varying clinical guidelines, approval statuses of ICIs, and the availability of supportive therapies (42).

Regional subgroup analysis (comparing Asian populations with Western and South American populations) revealed interesting differences in the results. In the discussion, we should delve deeper into the reasons that may lead to these differences, such as genetic characteristics, environmental factors, or healthcare access in different populations, which may affect the efficacy of immune checkpoint inhibitors (ICIs) in different populations. The pooled hazard ratio (HR) for the Asian population is 1.75 (95% CI: 0.74-4.15), which is higher than the HR of 1.47 (95% CI: 1.06-2.04) for the Western population. However, there is high heterogeneity among the Asian population (I²=86%), indicating the need for further research to better understand the differences in outcomes within the region. This regional difference may be attributed to the diversity of genetic factors, differences in healthcare systems, or variations in treatment plans. For example, Western populations may have broader opportunities for early screening, more advanced treatment methods, and more opportunities to participate in clinical trials, all of which may affect overall treatment outcomes. At the same time, there may be differences in treatment plans and drug combinations in different regions, which further increases the complexity of the results. By comprehensively considering these factors, we can gain a more comprehensive understanding of the impact of regional differences on treatment outcomes.

In addition, the meta-analysis included subgroup analyses based on PD-L1 expression and the line of treatment (first-line versus second-line or beyond). Patients with high PD-L1 expression generally respond better to ICIs; however, the presence of bone metastasis appears to attenuate this benefit. In patients with high PD-L1 expression, ICIs such as pembrolizumab and nivolumab have demonstrated significant improvements in both overall survival (OS) and progression-free survival (PFS). However, the presence of bone metastasis diminishes the full extent of this benefit. Several studies have proposed that the immune suppressive microenvironment within the bone could limit the potential of ICIs, even in patients who are otherwise good candidates for these therapies based on PD-L1 expression levels. This highlights the need to explore whether additional therapies could overcome this immune suppression and enhance the effectiveness of ICIs in patients with bone metastasis (43–45).

### 4.4 Potential mechanisms of resistance in bone metastasis

The microenvironment of bone metastasis presents unique challenges that could explain the reduced efficacy of ICIs observed in this meta-analysis. As discussed earlier, bone metastasis creates a complex interplay between immune cells, tumor cells, osteoclasts, and osteoblasts. This interaction fosters an environment conducive to immune suppression, driven by factors such as transforming growth factor-beta (TGF-β), prostaglandins, and regulatory T cells (Tregs). These immunosuppressive factors may hinder the cytotoxic activity of T cells that ICIs seek to activate (46). Furthermore, the bone marrow microenvironment, which plays a crucial role in bone metastasis, contains a large number of hematopoietic and stromal cells that can contribute to immune evasion. Myeloid-derived suppressor cells (MDSCs) and Tregs are commonly found in the bone marrow and are known to suppress anti-tumor immune responses. This may explain why bone metastasis is particularly resistant to ICI therapy, as the immune system is unable to adequately target and destroy tumor cells within the bone microenvironment (47). Additionally, high tumor burden, which is often associated with the presence of bone metastasis, may lead to immune exhaustion. T cells become “exhausted” in the context of prolonged and high-intensity exposure to tumor antigens, resulting in reduced functionality and responsiveness to ICIs. The immunosuppressive nature of the bone microenvironment, combined with the effects of immune exhaustion, may explain the relatively poor outcomes observed in patients with bone metastasis treated with ICIs (48).

The findings from this meta-analysis have important clinical implications for the management of NSCLC patients with bone metastasis. Given that bone metastasis is associated with poorer survival outcomes, clinicians may need to adopt more aggressive treatment strategies or consider alternative therapeutic combinations to enhance the efficacy of ICIs in this subgroup of patients. For example, the combination of ICIs with other therapies, such as bisphosphonates, denosumab, or radiation therapy, may help mitigate the immunosuppressive effects of bone metastasis. Bisphosphonates and denosumab are commonly used to manage bone metastasis by inhibiting osteoclast activity and reducing skeletal-related events (SREs). Preclinical studies have suggested that these agents may also have immune-modulating effects, potentially enhancing the efficacy of ICIs. For example, denosumab has been shown to reduce the immunosuppressive activity of Tregs and MDSCs in the tumor microenvironment, which may allow for a more effective anti-tumor immune response when combined with ICIs. Similarly, the use of localized radiation therapy to treat bone metastasis could potentially prime the immune system by inducing immunogenic cell death, thereby enhancing the response to ICIs.

The need for a tailored treatment approach is particularly relevant for patients with high PD-L1 expression or those who are candidates for first-line ICI therapy. While ICIs have demonstrated significant benefits in these populations, the presence of bone metastasis may necessitate adjustments to the standard treatment protocol. Further clinical trials are needed to explore the potential benefits of combining ICIs with other therapeutic agents in this specific patient subgroup (49).

### 4.5 Limitations of the study

While this meta-analysis provides valuable insights into the impact of bone metastasis on NSCLC patients treated with ICIs, several limitations should be considered when interpreting the results. First, there was substantial heterogeneity across the included studies, particularly in the Asian subgroup. This heterogeneity could be attributed to differences in study design, patient populations, treatment protocols, and genetic factors. While the random-effects model was used to account for this variability, the results should be interpreted with caution, especially in subgroups with high heterogeneity. Second, the meta-analysis relied on data extracted from published studies, and there may have been variations in how bone metastasis was defined and diagnosed across studies. This could have introduced bias, as some studies may have included patients with different extents of bone metastasis or different criteria for disease progression. Moreover, data on skeletal-related events (SREs) and their impact on survival outcomes were not consistently reported across studies, limiting the ability to assess the full extent of bone metastasis-related complications.

### 4.6 Future directions

The findings of this meta-analysis hold significant clinical implications for the treatment of NSCLC patients with bone metastasis. While immune checkpoint inhibitors (ICIs) have revolutionized the treatment of NSCLC, the presence of bone metastasis is associated with significantly worse overall survival (OS) and progression-free survival (PFS) outcomes. This suggests that clinicians should carefully evaluate the treatment approach for this specific patient population.

Despite the poorer prognosis, ICIs should still be considered a key treatment option, particularly in patients with high PD-L1 expression or those who are not candidates for other therapies. However, the evidence suggests that ICIs may not be as effective in patients with extensive or aggressive bone metastasis due to the immunosuppressive microenvironment within bone tissue. This raises the question of whether ICI monotherapy is sufficient for these patients or if a more comprehensive treatment strategy is needed. Given the challenges associated with treating NSCLC patients with bone metastasis, combining ICIs with other therapies could offer a more effective approach. For example, the use of bone-targeted agents like bisphosphonates or denosumab could help manage skeletal-related events (SREs) while potentially enhancing the immune response, making ICIs more effective. Additionally, the use of localized treatments such as radiation therapy to bone metastases may not only alleviate symptoms but also improve ICI efficacy by inducing immunogenic cell death and stimulating the immune system.

Furthermore, emerging evidence supports the combination of ICIs with chemotherapy or anti-angiogenic therapies, which may provide synergistic effects that improve both systemic control of the disease and localized treatment of bone metastases. Clinicians should consider these combination approaches, particularly for patients with a high tumor burden or extensive bone involvement, as a more comprehensive strategy may improve survival outcomes. Ultimately, these findings highlight the need for a more personalized approach to treating NSCLC patients with bone metastasis. Clinicians should carefully consider the extent of metastatic disease, the patient’s overall health status, and PD-L1 expression when deciding on the most appropriate treatment plan. Early integration of bone-modifying agents and close monitoring of disease progression may improve outcomes and quality of life for these patients.

## 5 Conclusion

In conclusion, this systematic review and meta-analysis demonstrate that bone metastasis is a strong negative prognostic factor for NSCLC patients treated with ICIs. The presence of bone metastasis is associated with significantly worse overall survival and progression-free survival outcomes, underscoring the need for tailored treatment approaches for this patient population. While ICIs have transformed the treatment landscape of NSCLC, the challenges posed by bone metastasis require further research and innovative therapeutic strategies to optimize outcomes for these patients. Future studies should focus on identifying biomarkers of response, exploring combination therapies, and addressing the underlying mechanisms of immune resistance in the bone microenvironment.

## Data Availability

The original contributions presented in the study are included in the article/supplementary material. Further inquiries can be directed to the corresponding authors.
